# The Functional Significance of Common Polymorphisms in Zinc Finger Transcription Factors

**DOI:** 10.1534/g3.114.012195

**Published:** 2014-06-26

**Authors:** Sarah H. Lockwood, Anna Guan, Abigail S. Yu, Chi Zhang, Artem Zykovich, Ian Korf, Bruce Rannala, David J. Segal

**Affiliations:** * Genome Center and Department of Biochemistry and Molecular Medicine, University of California, Davis, California 95616; † Genome Center and Department of Evolution and Ecology, University of California, Davis, California 95616; ‡ Genome Center and Department of Molecular and Cellular Biology, University of California, Davis, California 95616

**Keywords:** *trans*-expression quantitative trait loci, zinc finger proteins, nonsynonymous SNPs, Hardy-Weinberg Equilibrium, transcription factors

## Abstract

Variants that alter the DNA-binding specificity of transcription factors could affect the specificity for and expression of potentially many target genes, as has been observed in several tumor-derived mutations. Here we examined if such *trans* expression quantitative trait loci (*trans*-eQTLs) could similarly result from common genetic variants. We chose to focus on the Cys_2_-His_2_ class of zinc finger transcription factors because they are the most abundant superfamily of transcription factors in human and have well-characterized DNA binding interactions. We identified 430 SNPs that cause missense substitutions in the DNA-contacting residues. Fewer common missense SNPs were found at DNA-contacting residues compared with non-DNA-contacting residues (*P* = 0.00006), consistent with possible functional selection against SNPs at DNA-contacting positions. Functional predictions based on zinc finger transcription factor (ZNF) DNA binding preferences also suggested that many common substitutions could potentially alter binding specificity. However, Hardy-Weinberg Equilibrium analysis and examination of seven orthologs within the primate lineage failed to find evidence of *trans*-eQTLs associated with the DNA-contacting positions or evidence of a different selection pressure on a contemporary and evolutionary timescales. The overall conclusion was that common SNPs that alter the DNA-contacting residues of these factors are unlikely to produce strong *trans*-eQTLs, consistent with the observations by others that *trans*-eQTLs in humans tend to be few and weak. Some rare SNPs might alter specificity and remained rare due to purifying selection. The study also underscores the need for large-scale eQTLs mapping efforts that might provide experimental evidence for SNPs that alter the choice of transcription factor binding sites.

Genetic variation in humans influences many traits, including development and susceptibility to disease (Stranger *et al.* 2007b; Schadt *et al.* 2008; Gibbs *et al.* 2010; Montgomery *et al.* 2010). Common single nucleotide polymorphisms (SNPs), those occurring in 1% or more of a population, can be grouped into two broad categories based on their relationship to the gene they affect. SNPs that change the expression of the gene in which they occur, such as by altering a coding exon or promoter binding site, are considered *cis* expression quantitative trait loci (*cis*-eQTLs). Those exerting an effect on a different gene are considered *trans* expression quantitative trait loci (*trans*-eQTLs). SNPs in nongeneic regions such as enhancers are also considered *trans*-eQTLs if they alter expression of a gene that is more than 100 kb away (Stranger *et al.* 2007a). The *trans*-eQTLs have been observed less frequently than *cis*-eQTLs in humans and tend to display reduced effects on the regulated gene (Schadt *et al.* 2008). The mechanisms have also been less well-studied. Such SNPs could alter the sequence of distal enhancer elements, change the expression level of a regulatory RNA or protein, or, in principle, alter the DNA-binding specificity of a transcription factor and thus change its selection of target genes. Because transcription factors often have multiple target genes, SNPs of this latter class might be predicted to alter the expression of many genes.

In this study, we examined the hypothesis that common SNPs in transcription factors can lead to changes in the spectrum and expression level of target genes. We chose to focus on the Cys_2_-His_2_ (C2H2) class of zinc finger transcription factors (ZNFs) because they are the most abundant superfamily of transcription factors in human (more than 700 members), accounting for nearly half of all annotated transcription factors (Tupler *et al.* 2001; Vaquerizas *et al.* 2009). In addition, although the ability to predict their DNA binding preferences is far from perfect, more is known about ZNF DNA recognition than for any other class of human transcription factors. Thus, they represent the best possibility to predict the effect of SNP-dependent alterations in target gene recognition (Wolfe *et al.* 2000).

The DNA-binding domain of ZNFs contains tandem arrays of zinc finger repeats or “fingers.” Arrays of up to 40 fingers have been reported (Wolfe *et al.* 2000), although typically only three to five fingers are directly involved in DNA binding. Each finger binds three to four base pairs of DNA. Sequence recognition is determined largely by interactions between the DNA bases and four amino acids in the zinc finger α-helix, namely those at positions −1, 2, 3, and 6 (Pavletich and Pabo 1991; Elrod-Erickson *et al.* 1996). Engineered substitutions of amino acids at these positions cause altered DNA binding specificity (Segal *et al.* 1999; Dreier *et al.* 2001), and rare natural mutations have been reported to alter the spectrum of genes targeted by the ZNF. Mutations in the zinc fingers of GFI1 were linked to severe congenital neutropenia (Person *et al.* 2003). Tumor-derived mutations in the DBDs of the tumor suppressor protein p53 (Campomenosi *et al.* 2001; Inga *et al.* 2001; Malcikova *et al.* 2010), thyroid hormone receptor (Chan and Privalsky 2009), and CTCF (Filippova *et al.* 2002) all resulted in altered DNA recognition and target gene selection.

Here we identified 430 SNPs that cause nonsynonymous substitutions in the four primary DNA-contacting amino acids in 252 ZNFs in the human genome. Evidence of selection against common SNPs at DNA-contacting compared to non-DNA-contacting amino acids supported our hypothesis that common variants in DNA-contacting positions could affect transcription factor function. However, we failed to find evidence of *trans*-eQTLs associated with any of the DNA-contacting positions in this study. To better understand potential selection pressures on these SNPs, we performed a broad analysis of sequence variation across primate species and within the human population for a subset of seven ZNFs. Most ZNFs appeared to be under negative selection pressure; there was little evidence of positive selection. The analysis also revealed a complex landscape of variation and function, with a few SNPs likely to have high functional significance but with most having little effect. These results therefore add to our understanding and highlight the complexities of genetic variation and *trans*-eQTLs.

## Materials and Methods

### Localization of SNPs within zinc finger domains

The fingerFinder.pl Perl script identifies clusters of three C2H2 zinc finger domains with TGEKP-like linkers between the fingers. The search used HMMER version 2.3.2 (Sonnhammer *et al.* 1998) and the Pfam profile for C2H2 zinc fingers PF00096.16 (Schuster-Bockler *et al.* 2004). The HMM profile is embedded within fingerFinder.pl to maintain consistency in case of updates. Protein sequences were obtained from the Ensemble database corresponding to the GRCh37/hg19 genome assembly. SNPs from dbSNP version 136 were identified at the C2, −2, −1, 1, 2, 3, 5, and 6 positions in each finger using Perl script snpTOzf.pl. Both Perl scripts are available at www.genomecenter.ucdavis.edu/segallab/segallabsoftware. Only SNPs from the 1000 Genomes project (release 20110521) that also contained frequency data were retained for this study.

### Hardy-Weinberg Equilibrium analysis

For the 1040 DNA-contacting and non-DNA-contacting SNPs, chromosomal positions were obtained from Ensemble Biomart Variation 72. The VCFtools *htscmd* command (github.com/samtools/htslib) was used to extract diploid genotypes for all 2188 individuals from the variant call format (vcf) files of 1000 Genomes release 20110521. Individuals were then subsetted by population according to phase1_integrated_calls.20101123.ALL.panel. The allele and genotype frequencies for each SNP were computed with a custom R script. Deviation from Hardy-Weinberg Equilibrium (HWE) was assessed by applying both a chi-square test and a Fisher exact test. Calculations were not performed if any genotype category contained less than five counts. The *HWExact* command from the GWASExactHW R package (Painter and Washington 2013) was used to compute the Fisher exact test. *D* was computed by subtracting the expected number (assuming that the SNP is in HWE) from the observed number of heterozygous individuals divided by two. A positive *D* indicates the number of observed heterozygous individuals is greater than expected, suggesting that heterozygosity at the locus may be beneficial. A negative *D* suggests that one or both homozygotes were favored.

For analysis of the seven ZNFs, the genotypes of SNPs located in the coding sequences were extracted from the vcf files based on the genomic coordinates from NCBI’s Consensus CDS (CCDS) database (release 9; September 7, 2011). ZNF99 did not have a CCDS number; its genomic coordinates were obtained using BLAT to search the UCSC Genome Browser human genome (GRCh37/hg19). Classification of each SNP as missense, silent, or other (downstream variant, frameshift variant, gain/loss of stop codon) was obtained from the NCBI dbSNP database (version 136). The DNA contact status of any SNP not occurring in zinc finger positions −1, 2, 3, and 6 (DNA-contacting) or C2, −2, 1, and 5 (non-DNA-contacting) was designated as unknown.

### Selection (dN/dS) analysis of seven zinc fingers in the primate lineage

For the human ortholog of the ZNFs, the longest transcript was chosen from the Nucleotide database of the National Center for Biotechnology Information (NCBI). The other primate orthologs of these ZNFs were obtained by performing a TBLASTN, an NCBI translated nucleotide database, with the human protein ortholog sequence against the Nucleotide Collection (nr/nt) database with a filter for primates (taxid:9443). The sequence with the highest blast score was retrieved for each primate. If the ZNF sequences for a particular primate had excessive missing data, then the primate was excluded from the study for that ZNF. Multiple alignments of the ortholog nucleotide and protein sequences were generated using TranslatorX (translator.co.uk) with the default setting, using Muscle as the protein alignment method (Abascal *et al.* 2010).

Likelihood ratio test was used to compare the model of neutral evolution (M1a) and that of positive selection (M2a). The site-specific model assumes all lineages share the same *ω* (*d*N/*d*S ratio) for each codon or amino acid site in the protein, but *ω* can vary among sites. The null model (M1a) assumes no positive selection. A proportion *p*_0_ of amino acids have *ω*_0_ < 1 (under negative selection) and the remaining proportion *p*_1_ = 1 − *p*_0_ have *ω*_1_ = 1 (are neutral). The alternative model (M2a) has one more class, having proportion *p*_2_ = 1 − *p*_0_ − *p*_1_ of amino acids with *ω*_2_ > 1 (positive selection). The χ^2^ distribution with two degrees of freedom was used for the likelihood ratio test. The species tree, obtained from the Tree of Life website for the primates (tolweb.org/Primates/15963/1999.01.01), was used as the guide tree for the codeml program in the PAML package (version 4.5) (Yang 2007). The Bayes Empirical Bayes (BEB) method was used to calculate the posterior probability of *ω* falling into each of the three classes: *ω* < 1, *ω* = 1, and *ω* > 1 (Yang *et al.* 2005).

### Functional and DNA-binding specificity predictions

For functional predictions, the database of nonsynonymous functional predictions (dbNSFP, version 2.0; release February 25, 2013) (Liu *et al.* 2011) was downloaded from sites.google.com/site/jpopgen/dbNSFP. The database was developed for functional prediction of all potential nonsynonymous single-nucleotide variants in the human genome and compiles prediction scores from prediction algorithms including SIFT (Human_db_37_ensembl_63), Polyphen2 (v2.2.2), MutationTaster (retrieved 2012), and FATHMM (v2.1). The database was queried for the 1040 SNPs using the java *search_dbNSFP20* command. Following the guidance of dbNSFP, a SNP was considered deleterious if it had a SIFT score <0.05, Polyphen2 HDIV score >0.95, a MutationTaster score >0.90, or FATHMM score <−1.5.

For DNA-binding specificity predictions, sequence logos and position frequency matrices (PFM) were obtained by entering the reference and SNP individual zinc finger sequences to the ZFModels website (stormo.wustl.edu/ZFModels) (Gupta *et al.* 2014) using the parameters ZF Protein Sequences, One Finger Model, and Information Content. The difference between the “REF” PFM (*R*) and the “SNP” PFM (*S*) was calculated as the Kullback-Leibler distance (*D*) for all positions (*i*) in the matrix as D(R||S) = ∑(R_i_ * ln(R_i_/S_i_)), as well as the reciprocal D(S||R) = ∑(S_i_ * ln(S_i_/R_i_)). The two distances were then added to create the summed Kullback-Leibler distance. Of the 435 REF and SNP fingers analyzed, ZFModels was unable to calculate PFMs for 19, which appear in Supporting Information, Table S1 as NA.

## Results

### Common missense SNPs affecting the DNA-contacting residues of zinc finger proteins are less abundant than those at non-DNA-contacting residues

**Figure 1:**
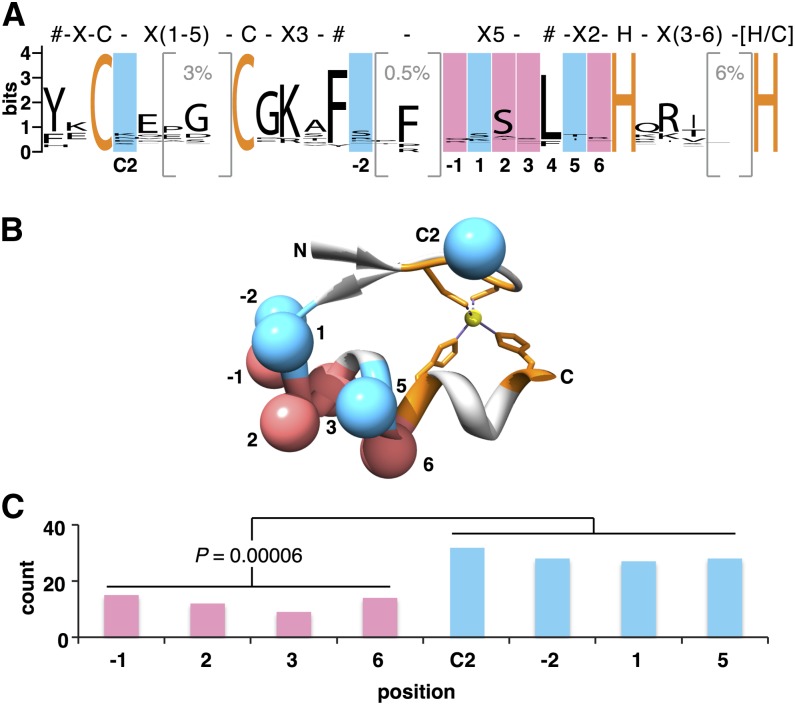
Common SNPs (MAF ≥1%) are observed significantly less frequently at DNA-contacting than at non-DNA-contacting amino acids. (A) The sequence logo representing the 3415 human zinc fingers in this study. The PFAM description of a C2H2 zinc finger motif (PF00096) is shown above the logo. # indicates amino acid positions important for the structure of the motif. Conserved Cys and His residues are indicated in orange. Bracketed regions represent additional amino acids observed in a minority of fingers (percent indicated in gray). Position numbering, by convention starting at the first amino acid of the α-helix, is shown below the logo. (B) The structure of a typical C2H2 zinc finger (Finger 2 of Zif268, PDB accession AAY1) with conserved Cys and His residues (orange) ligating a zinc ion (yellow). The β carbons (spheres) of residues on the DNA-contacting face of the finger (red) point down toward the DNA bases (not shown), whereas non-DNA-contacting residues (blue) face away from the DNA. (C) The number of common SNPs (as defined in *Materials and Methods*) at DNA-contacting (red) and non-DNA-contacting (blue) residues of the zinc finger.

C2H2 zinc fingers are known to mediate DNA as well as RNA and protein interactions (Mackay and Crossley 1998; Brown 2005). To maximize the likelihood of examining fingers that bind DNA in all potential splice isoforms, the human proteome was searched for clusters of three or more fingers joined by TGEKP-like linkers. In mammals, zinc fingers are typically found in tandem arrays, with approximately 50% of fingers connected by linkers having the sequence TGEKP (Wolfe *et al.* 2000). Almost every residue in this conserved linker plays an identifiable role in stabilizing the protein–DNA interaction. Although some known DNA-binding ZNFs do not have this linker (*e.g.*, Tramtrack ZF1-2) and some that do not bind DNA do have it (*e.g.*, Gli ZF2-3), an array of two to three fingers joined by a TGEKP-like linker is currently the best predictor of DNA binding (Ryan and Darby 1998; Brayer *et al.* 2008). Only SNPs causing missense mutations at DNA-contacting positions −1, 2, 3, and 6 or non-DNA-contacting positions C2, −2, 1, and 5 were retained for this study. SNPs causing frameshift or splice mutations were omitted to avoid changes in ZNF specificity due to truncations or loss of entire exons. The ability of the zinc fingers to recognize different DNA sequences is due to the diversity of the amino acids that appear in the DNA-contacting positions. Thus, when zinc fingers that recognize different DNA sequences are aligned, these positions appear to have little conservation (Figure 1A). For comparative purposes, non-DNA-contacting positions were chosen that had similarly low sequence conservation. However, in contrast to the DNA-contacting residues, positions C2, −2, 1, and 5 are thought to have low conservation because they have no functional role in DNA recognition or protein folding (Wolfe *et al.* 2000). In particular, beta carbons of these residues direct the side chains away from the DNA bases, unlike the DNA-contacting residues that point directly at the bases (Figure 1B). The search identified 1040 missense SNPs in 398 ZNF proteins (Table S1). Of these, 166 SNPs were “common” [minor allele frequency (MAF) >1%] with respect to the combined 2188 individuals of the 1000 Genomes dataset. Significantly fewer common SNPs were found in the DNA-contacting positions compared with the non-DNA-contacting positions (*P* = 0.00006) (Figure 1C). One interpretation is that substitutions of the DNA-contacting amino acids altered the DNA-binding specificity of the protein, leading to deleterious effects and negative selection.

### No *trans*-eQTLs have been reported for missense SNPs at the DNA-contacting positions

SNPs that alter DNA-binding specificity might cause the protein to regulate a different spectrum of target genes. The SNP could cause the loss or gain of affinity to a promoter or enhancer of one or more target genes that are distant to the gene encoding the transcription factor, thus producing *trans*-eQTLs. The NCBI maintains the GTEx (Genotype-Tissue Expression) eQTL Browser (www.ncbi.nlm.nih.gov/gtex) that is currently based on gene expression data from liver, brain, and lymphoblastoid cell lines (Stranger *et al.* 2007b; Schadt *et al.* 2008; Gibbs *et al.* 2010; Montgomery *et al.* 2010). Only four of the 1040 SNPs were reported to be associated with eQTLs at a significance less than 10^−5^ (Table 1). Interestingly, all four produced *trans*-eQTLs, all were common SNPs, and all were in non-DNA-contacting positions of the ZNFs. Three were found to deviate from HWE (described below), which in all cases favored a heterozygous state (in contrast, 85% of deviating SNPs in this study favored a homozygous state). The potential significance of these *trans*-eQTLs associated with non-DNA-contacting regions is explored in the *Discussion*. However, no *cis* or *trans*-eQTLs were reported for any SNPs at the DNA-contacting positions. Two additional studies examining gene expression in skin, adipose, lymphoblastoid cell lines, and peripheral blood did not report *cis* or *trans*-eQTL for any of the 1040 SNPs (Fehrmann *et al.* 2011; Grundberg *et al.* 2012).

**Table 1:** eQTLs reported in the literature for the 1040 missense SNPs (*P* < 1.0E−5)

Finger Position	SNP		Probe		Distance (bp)	Effect	*P*	Tissue	Study
	ID	Gene	ID	Gene					
C2	rs2230752*^a^*	ZNF177	GI_37622342-A	ZNF266	30,946	*trans*	2.0E−26	LBL	Stranger *et al.* 2007b
			ILMN_1753782	ZNF266	30,901	*trans*	7.0E−12	Cerebellum	Gibbs *et al.* 2010
C2	rs7257872*^a^*	ZNF584	GI_13325056-S	SLC27A5	81,182	*trans*	3.1E−11	LBL	Stranger *et al.* 2007b
−2	rs2074060	ZNF772	ILMN_1680693	ZNF419	13,513	*trans*	1.7E−09	Temporal cortex	Gibbs *et al.* 2010
							1.2E−08	Frontal cortex	Gibbs *et al.* 2010
5	rs1465789*^a^*	ZNF132	GI_13325056-S	SLC27A5	63,912	*trans*	3.2E−14	LBL	Stranger *et al.* 2007b
				ZNF132	1345	*cis*	7.8E−08	LBL	Stranger *et al.* 2007b

LBL, lymphoblastoid cell lines.

a Not in Hardy-Weinberg Equilibrium; observed heterozygotes more than expected.

### SNPs deviating from HWE are few and occur in both DNA-contacting and non-DNA-contacting positions

**Figure 2:**
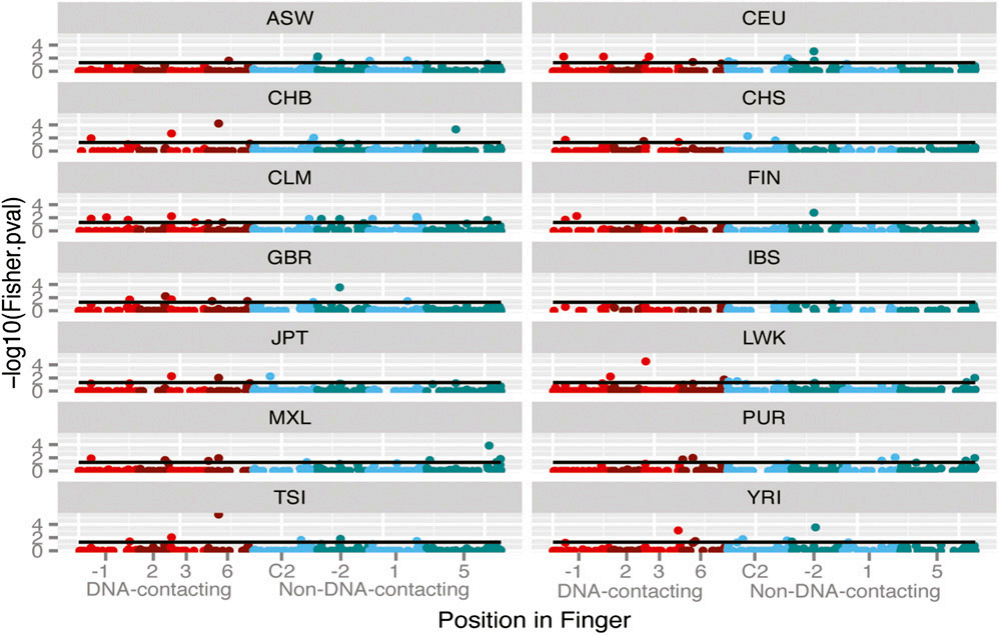
SNPs deviating from Hardy-Weinberg Equilibrium (HWE) for each of the 14 populations in the 1000 Genomes data set. The probability of deviation from HWE is shown for SNPs occurring in DNA-contacting (light and dark red) and non-DNA-contacting (light and dark blue) positions. The black horizontal line in each graph corresponds to a Fisher exact *P* value of 0.05.

One explanation for the failure to observe *trans*-eQTLs for the altered DNA-contacting positions is a simple lack of data. Very little is known about the biology of most ZNFs, and it is likely that they could exert their regulatory influence in specific cell types or developmental stages that were not examined in the six studies above. An alternative method to investigate if the missense SNPs have functional effects is to determine whether the genotype frequencies deviate from HWE. Deviations from HWE are caused by evolutionary influences such as selection, but also mutation, non-random mating, or recent population admixture. The effects of admixture can be reduced by confining examination to individual populations of the 1000 Genomes project. SNPs that deviate from HWE under these conditions are likely to represent either positive or negative selection. A high MAF may suggest positive selection; a low MAF may suggest negative selection. An excess of heterozygotes can indicate overdominance selection. However, if the MAF is too low, then there may be too few genotypes to accurately calculate HWE. Of the 1040 missense SNPs, only 55 (5%) demonstrated significant deviation from HWE (*P* < 0.05), and only 13 (1%) deviated from HWE in more than one of the 1000 Genomes populations (Figure 2 and Table S1). Of the 55 deviants, 85% had a negative *D* value, indicating selection favoring the homozygous state for most SNPs. However, after adjusting for the total number of SNPs, there was no significant difference in the percent of SNPs deviating at DNA-contacting or non-DNA-contacting positions (*P* = 0.64).

### Orthologs in the primate lineage reveal a trend of negative selection

**Figure 3:**
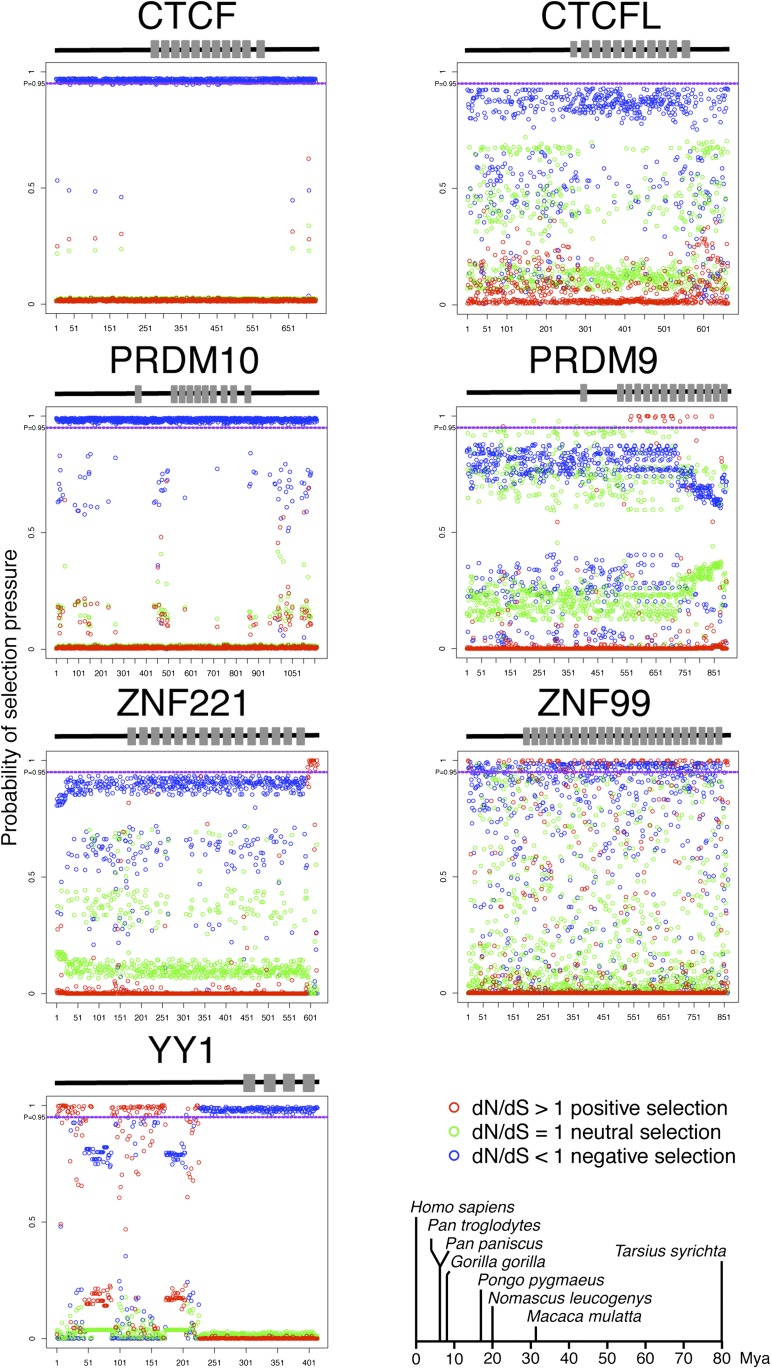
Selection pressures on zinc finger orthologs within the primate lineage. The posterior probability of positive (red), neutral (green), or negative (blue) selection pressure at each amino acid position (x-axis) based on the Bayes Empirical Bayes (BEB) analysis of orthologs in the primate lineages are shown for seven zinc finger proteins. Gray bars on the cartoon above each graph indicate the positions of zinc fingers within the protein. The dashed purple horizontal line at the top of each graph corresponds to 95% probability. The approximate evolutionary distances, in millions of years ago [Mya; based on (Perelman *et al.* 2011)], of the eight species used in this analysis are shown at the lower right.

Evidence for functional selection based on genetic variation within the human population can be potentially confounded by several factors. Many variants in the human genome may have arisen relatively recently due to rapid population expansion and therefore may not have had sufficient time for selection. Also, selection may be most important in the early stages of species evolution. To increase the power of our test for selection, we examined orthologs that spanned the evolutionary time periods between species in the primate lineage. Orthologs of human CTCF, CTCFL, PRDM10, PRDM9, YY1, ZNF221, and ZNF99 were found (see *Materials and Methods*) in the translated genomes of *Pan troglodytes* (chimpanzee), *Pan paniscus* (bonobo or pygmy chimpanzee), *Gorilla gorilla gorilla* (gorilla), *Pongo pygmaeus abelii* (orangutan), *Nomascus leucogenys* (gibbons), *Macaca mulatta* (rhesus macaque), and *Tarsius syrichta* (tarsier). To identify amino acid sites undergoing positive selection, the codeml program was used to perform a likelihood ratio test between the null model of neutral evolution (M1a) and alternative model of positive selection (M2a). The test statistics follows a chi-square distribution of degree 2. The nonsynonymous-to-synonymous rate ratio, *ω* (*d*N/*d*S), measures selective pressure at the protein level. A site undergoing positive selection can be inferred when *ω* is greater than 1. The *P* values of the likelihood ratio tests are 0.9995, 1.00, 0.904, 0.000, 0.000, 0.000, and 0.000 for CTCF, CTCFL, PRDM10, PRDM9, YY1, ZNF221, and ZNF99, respectively. These are consistent with the red dots above the *P* = 0.95 lines in Figure 3. The Bayes Empirical Bayes (BEB) method was used to calculate the posterior probability of *ω* falling into the three classes: *ω* < 1, *ω* = 1, and *ω* > 1 (Yang *et al.* 2005). The differences in selective pressure between this set of proteins were quite striking (Figure 3). However, the general observation was that almost all positions in the zinc fingers showed *ω* < 1, indicating negative selection. Significant (posterior probability >0.95) negative selection was observed for the zinc fingers of CTCF, PRDM10, ZNF99, and YY1 (the last half sites), whereas those of CTCFL, PRDM9, and ZNF221 did not display strong evidence of negative selection. Two notable deviations were several positions in the fingers of PRDM9 and ZNF99 that showed significant evidence for positive selection (Figure 3, red dots above probability 0.95). However, for zinc fingers displaying negative selection, essentially all the residues are likely functionally important, and missense SNPs such as those in this study would likely not be tolerated.

### Missense SNPs at DNA-contacting positions are generally not predicted to be deleterious but might change DNA-binding specificity

**Figure 4:**
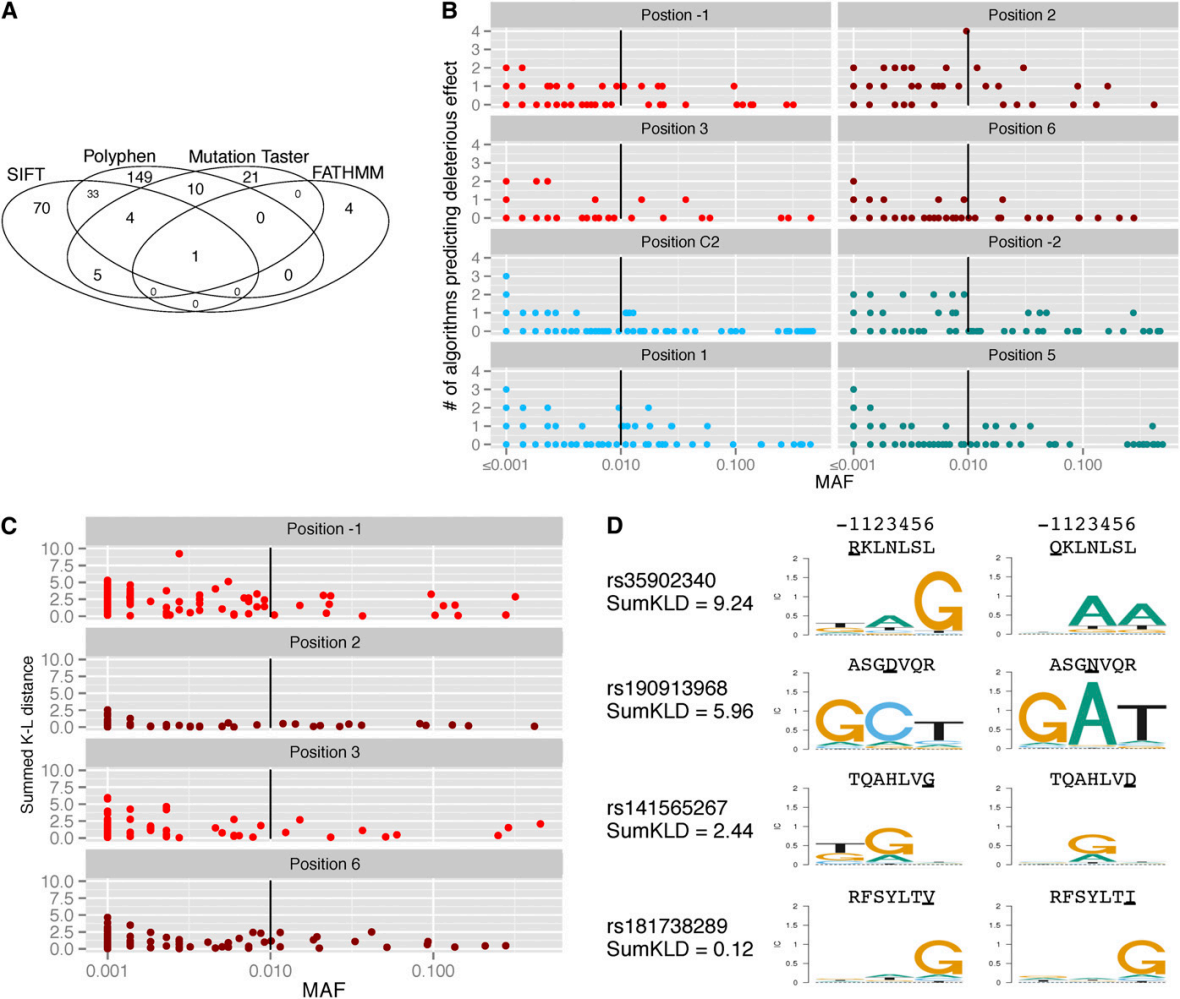
Predictions of deleterious and altered DNA sequence recognition effects of missense SNPs. (A) The overlap of predictions made by four algorithms that determine if a SNP will have a deleterious effect on protein function. (B) The confidence of the predictions (*i.e.*, an increased number of algorithms making the prediction) is shown as a function of the MAF. SNPs occurring in DNA-contacting (light and dark red) and non-DNA-contacting (light and dark blue) positions are shown. The black vertical line corresponds to MAF of 1%. The frequency of SNPs occurring at less than 0.01% in the 1000 Genomes populations cannot be accurately quantified; therefore, such SNPs are clustered as MAF ≤0.001. SNPs with variable low MAFs may be overlapping in these columns. (C) The summed Kullback-Leibler distance between the DNA binding sites of the reference and SNP forms of the affected zinc finger, as predicted by ZFModels (Gupta *et al.* 2014), is shown as a function of the MAF. Larger values indicate grater divergence. (D) Representative sequence motifs for several summed Kullback-Leibler distances. The amino acid changed by the SNP is underlined. Note that the actual protein–DNA interactions are inverted in this depiction; the amino acid in position −1 typically affects the most 3′ base and position 6 typically affects the most 5′ base.

The low number of SNPs deviating from HWE and the lack of difference between DNA-contacting and non-DNA-contacting positions suggest that the majority of polymorphisms in the DNA-contacting amino acids do not produce an effect that is either beneficial or deleterious to fitness. There are many algorithms to predict if nonsynonymous variants might lead to deleterious effects on protein function. For example, dbNSFP 2.0 is an integrated database of functional annotations from multiple sources for the comprehensive collection of more than 87,361,054 human nonsynonymous SNPs. It compiles scores from prediction algorithms such as SIFT (Kumar *et al.* 2009), Polyphen2 (Adzhubei *et al.* 2010), MutationTaster (Schwarz *et al.* 2010), and FATHMM (Shihab *et al.* 2013). Because each algorithm used a somewhat different set of criteria to predict if a SNP would be deleterious, we decided to place increasing confidence in SNPs predicted by multiple methods. Interestingly, there was very little agreement among methods regarding which of the 1040 SNPs might be functionally deleterious. Of the 314 SNPs predicted to be deleterious, only one was common to all four methods (Figure 4A and Table S1). With rare exceptions, common SNPs were not predicted to be deleterious or were predicted to be so by only one method (Figure 4B). SNPs predicted to be deleterious by two, three, or four methods usually had MAFs <0.1, in general agreement with the concept that truly deleterious alleles tend to be rare in populations due to negative selection. However, there were no notable differences between DNA-contacting and non-DNA-contacting positions in the number or distribution of predicted deleterious alleles, again suggesting that SNPs in the DNA-contacting positions are not more likely to disrupt the function of the protein than SNPs at any other position.

However, a SNP that changes DNA-binding specificity may not necessarily be expected to alter protein function. The more likely expectation would be that the transcription factor would still bind, but to somewhat different DNA targets. Unfortunately, it is currently not possible to accurately predict the target sites for any DNA-binding protein based only on its primary sequence (with the possible exception of transcription activator–like effector proteins, which are not found in the human genome) (Boch *et al.* 2009; Moscou and Bogdanove 2009). Considerable progress has also been made in predicting the binding behavior of ZNF proteins, based on large datasets of natural and engineered zinc fingers (Gupta *et al.* 2012; Enuameh *et al.* 2013; Zhu *et al.* 2013; Gupta *et al.* 2014; Persikov *et al.* 2014; Persikov and Singh 2014). A recently described random forest-based predictive model, ZFModels (Gupta *et al.* 2014), was used to estimate the specificity of individual zinc fingers that harbor a SNP. The summed Kullback-Leibler distance was used to quantify the difference between the predicted DNA binding sites of the reference and SNP forms of the zinc finger. The more dramatic changes in predicted specificity (summed KL >2.5) were found for SNPs with MAFs <0.1 (Figure 4, C and D). These data suggest that some of the SNPs, particularly rare SNPs, would be expected to alter the DNA-binding specificity of their ZNFs.

## Discussion

The role of natural genetic variation in human health and disease has been a major area of focus in the past several years. Most variants act in *cis* to the genes they affect, often altering promoter regions, coding regions, or splice sites (Stranger *et al.* 2007a,b). Here we have investigated a potential mechanism for how genome-encoded information could affect gene expression in *trans*. We hypothesized that natural genetic variation could alter the binding site preferences and activities of ZNF transcription factors and therefore alter their gene regulatory functions. In the context of cancer, some tumor-specific mutations in transcription factors had been reported to alter DNA-binding specificity, providing proof-of-concept (Campomenosi *et al.* 2001; Inga *et al.* 2001; Filippova *et al.* 2002; Chan and Privalsky 2009; Malcikova *et al.* 2010). Our initial observation in this study that there are significantly less *common* SNPs at the DNA-contacting positions compared with non-DNA-contacting positions encouraged us that rare SNPs may have functional consequences, and that selective pressure is operating against them.

Functional consequences could include the transcription factor binding either better or worse to its original target sites, or a change in specificity such that some new gene targets are regulated. In most cases, these consequences should be manifest in the change in expression of one or more genes that was dependent on the SNP in the ZNF gene, that is, *trans*-eQTLs. It is important to note that not all C2H2 zinc finger proteins are transcription factors and thus would not be expected to associate with eQTLs. For example, PRDM9 (Baudat *et al.* 2013) and CTFC (Ong and Corces 2014) would manifest altered DNA recognition as a change in homologous recombination hotspots and chromatin looping boundaries, respectively. However, despite decades of research of engineered zinc fingers, approximately 90% of naturally occurring ZNFs remain largely unstudied. Little is known about where these proteins bind or which genes they regulate. Databases of transcription factor binding motifs, such as TRANSFAC (Matys *et al.* 2006) and JASPAR (Mathelier *et al.* 2014), contain relatively few position weight matrices (PWMs) for ZNFs. For example, of the 252 ZNFs that had nonsynonymous substitutions in the four primary DNA-contacting amino acids in this study, only two were found in the JASPAR 2014 Core dataset of PWMs (jaspar.genereg.net). Furthermore, these databases do not provide information regarding which DNA-contacting amino acids are responsible for the observed PWM pattern. It is therefore not possible to accurately assign a position in the PWM to an amino acid affected by a SNP.

In principle, changes in transcription factor binding or gene expression could be measured directly *in vitro* or in cell culture with exogenously expressed proteins. However, these experiments are technically challenging for most zinc finger proteins. The mean number of zinc finger repeats in human proteins is 8.5, but some proteins contain 30 or more zinc finger repeats (Emerson and Thomas 2009). Although most KRAB-ZNFs encode all fingers in one exon, many without KRAB, such as CTCFL and PRDM10, have more than seven isoforms that typically splice together different sets of fingers. Engineering the mutant allele is laborious, and purification for *in vitro* studies is problematic because the two critical cysteine residues on each zinc finger quickly oxidize and lose binding activity. Overexpression of natural ZNFs in cells is often cytotoxic, and nonphysiological concentrations can lead to occupancy of atypical binding sites.

For these reasons, we chose instead to use existing data from large-scale studies of natural eQTLs in various cell types. Unfortunately, these studies failed to identify eQTLs for any of the SNPs in DNA-contacting positions. These results could indicate that the SNPs do not result in altered expression of any gene. However, the power of detection might be limited. In humans, *trans*-eQTLs are far less frequent than *cis*-eQTLs, and their effect sizes are typically small (Stranger *et al.* 2007b; Schadt *et al.* 2008; Gibbs *et al.* 2010; Montgomery *et al.* 2010). Although the large number of SNP-containing proteins in our study (398 ZNFs out of a total of approximately 712 in the genome) should have ensured that at least some would be expressed in the cell types available, little is known about the cell types and developmental stages in which these proteins are normally expressed. Also, the effect on gene expression may have been too small to detect, especially given the adjustments for multiple testing required in a genome-wide survey. The results from the other functional prediction experiments in this study could form the basis of a more targeted approach for seeking *trans*-eQTLs in the future.

Interestingly, the only gene expression effects that were observed were *trans*-eQTLs at the non-DNA-contacting positions. Of the four ZNFs containing such SNPs, *ZNF584* and *ZNF132* affected expression of the same gene, *SLC27A5*. The EMBL–EBI interaction database IntAct only found data for ZNF177, which also indicated an interaction with SLC27A5. Although it is not completely clear how alterations to non-DNA-contacting residues could alter the expression of a gene in *trans*, one hypothesis is that these fingers may actually facilitate protein–protein rather than protein–DNA interactions. Furthermore, the three ZNFs show significant deviation from HWE, and in the unusual direction of favoring the heterozygous genotype. It could be that ZNF177, ZNF584, and ZNF132 form a complex with the SLC27A5 gene or gene product to regulate its transcription. It is known that some C2H2 zinc fingers can bind RNA or protein, and that the protein interaction can involve any face of the finger (Brayer and Segal 2008), including the β-turn (position C2), the loop (position -2), and the α-helix (position 5). However, it cannot be ruled out that these non-DNA-contacting positions are influencing the neighboring DNA-contacting positions and exerting their effects by a DNA-recognition mechanism.

Having been unable to demonstratefunctional consequences for the SNPs, we sought evidence for selective pressure operating against the SNPs. HWE analysis revealed that very few missense SNPs were under selective pressure in the human genome, whereas the phylogenetic data suggested that missense SNPs should be under strong negative selection. The latter result was consistent with a previous study that also reported high conservation in all α-helix residues in cow-human-mouse ortholog trios (Emerson and Thomas 2009). One model that would be largely consistent with our seemingly opposing results is that common SNPs that could change DNA-binding specificity were likely selected against during human evolution, so that only SNPs that do not cause significant changes in binding specificity remain common today. This model would also be consistent with the relative paucity of common SNPs at DNA-contacting positions compared with non-DNA-contacting positions, the lack of robust prediction that they are deleterious, and the result that no eQTLs were found. The few common SNPs that deviate from HWE could be relatively new variants that arose at a frequency similar to SNPs in other non-DNA-contacting regions of the protein.

If the SNPs that alter DNA binding have been suppressed by negative selection, what is it about the remaining SNPs that would make them not functional? The majority of the common SNPs in this study were predicted to produce only modest changes in binding specificity (summed Kullback-Leibler <2.5). This change in an individual finger might be insufficient to alter the overall specificity of a multi-finger protein. It could also be that the individual finger was not used for DNA binding in that particular protein, and thus its substitution had no effect. This model may appear to discount the value of the phylogenetic data indicating that the amino acids from *Tarsius* to *Homo sapiens* were generally under negative selection. However, the majority of the data still support the model that many substitutions would not be tolerated, but the ones that persisted into the present are those that could be tolerated.

In summary, we report that common SNPs seem depleted in the DNA-contacting positions of ZNFs, but we find no significant evidence of function or selective pressure for those that remain. These data argue against our initial hypothesis that *common* SNPs in transcription factors might function as *trans*-eQTLs in the human genome. However, *rare* SNPs are more likely to deviate from HWE, to be predicted as deleterious, and to produce high-confidence DNA specificity changes. Algorithms that predict the deleterious nature of nonsynonymous mutations are gaining value in genome interpretation, especially in medicine. Such algorithms should consider this additional category of potentially nondeleterious but altered function. Further elucidation of the functional roles of these variants will be greatly aided by expanded large-scale eQTL mapping efforts such as the GTEx consortium.

## Supplementary Material

Supporting Information
