# Supplementary material for: The Functional Significance of Common Polymorphisms in Zinc Finger Transcription Factors
Source: G3 (Bethesda). 2014 Jun 26;4(9):1647–55. doi: 10.1534/g3.114.012195 (PMC4169156; doi:10.1534/g3.114.012195)
Supplement: Supporting Information [file supp_4_9_1647__index.html]

The Functional Significance of Common Polymorphisms in Zinc Finger Transcription Factors — Supporting Information 

# The Functional Significance of Common Polymorphisms in Zinc Finger Transcription Factors

## Supporting Information for Lockwood *et al.*, 2014

**Files in this Data Supplement:**

- Table S1 - Missense SNPs found at the indicated positions in human zinc finger genes. A complete list of all 1040 SNPs found in this study, including their position information, minor allele frequency (1000 Genomes), deviation from Hardy-Weinberg Equilibrium, prediction of deleterious function, and prediction of specificity change. (.xls, 299 KB)
